# An optimised promoter and signal peptide improves methionine production of a genetically engineered *Candida utilis* harboring the *δ-zein* gene

**DOI:** 10.3389/fmicb.2025.1586229

**Published:** 2025-07-04

**Authors:** Qiburi He, Shaofeng Su, Riqilang Ao, Lingli He, Xiao Wang, Mei Chun, Gaowa Gong

**Affiliations:** ^1^Inner Mongolia Academy of Agricultural and Animal Husbandry Sciences, Hohhot, China; ^2^Inner Mongolia Normal University, Hohhot, China; ^3^Hulunbuir Vocational Technical College, Hulunbuir, China

**Keywords:** *Candida utilis*, promoter, signal peptides, methionine, expression vector

## Abstract

**Introduction:**

L-methionine is nutritionally indispensable for humans and animals. It is widely applied to feed, livestock and poultry breeding, food, medicine, energy and chemical industries. Maize endosperm contains a stable protein called *δ-zein*, which is abundant in sulfur amino acids, including methionine. *Candida utilis* (*C. utilis*) has been utilized as a cell factory to express and produce recombinant products. However, there is limited information on its genetic background and expression regulatory elements.

**Methods:**

In this study, we aimed to improve methionine yields in an engineered *C. utilis* harboring the *δ-zein* gene by identifying a strong promoter and optimal signal peptide. A *C. utilis* glyceraldehyde-3-phosphate dehydrogenase (*GAP*) promoter mutant library was constructed and screened to obtain a strong promoter. Subsequently, *de novo* sequencing of the *C. utilis* genome was performed using a combination of second-generation Illumina-Seq sequencing platform and third-generation nanopore sequencing technique. Endogenous signal peptides of *C. utilis* were analyzed by sequencing the *C. utilis* genome. Recombinant *C. utilis* strains with homologous integration expression vectors of different signal peptides were constructed and screened for *C. utilis* optimal signal peptides for secretion of *δ-zein*.

**Results:**

Finally, a secretory expression system pGS-zein containing a strong promoter GP6 and an optimal signal peptide SP8 was constructed. In the food-grade engineered *C. utilis* C/pGS-zein methionine content increased by 21.09% compared with that of C/psP with the original promoter, and by 33.64% compared to wild-type *C. utilis*.

**Discussion:**

This study demonstrates successful expression and secretion of *δ-zein* in *C. utilis* and establishes a foundation for enhanced methionine production of heterologous proteins in *C. utilis*. More importantly, these high-performance biological elements provide fundamental knowledge and technical knowhow for enhanced production of heterologous proteins in *C. utilis*.

## 1 Introduction

Methionine is an essential component of proteins and functional molecular precursors to support human and animal nutrition. Methionine is required for growth, immunity, metabolism and other biological processes with important physiological significance. It is widely used in food, animal feed, medicine and other fields (Jankowski et al., 2014). Numerous human and animal model studies have shown that methionine is involved in the regulation of several functions *in vivo*, including cellular protein synthesis in the immune system (Lin et al., 2023), methylation reactions, redox, polyamine synthesis and coupling of folate metabolism (Shen et al., 2023). Methionine is an essential feed additive in animal husbandry and breeding as it is the main sulfur-containing amino acid in poultry and ruminant diets (Sałek et al., 2020). Methionine cannot be synthesized by livestock and must be obtained from ingestion of external sources. Methionine supplementation in feed can promote sufficient feed intake, which in turn, stimulates livestock growth, thus, minimizing losses and reducing production costs (Lee et al., 2020). Cardoso et al. showed that the addition of microbial proteins and rumen amino acids, such as methionine and lysine, into cereal based diets for dairy cows, achieved higher milk production and protein content (Cardoso et al., 2021). Barido et al. demonstrated that Met/Lys supplementation resulted in higher protein scores, water holding capacity and lower shear force score. Lower shear force typically signifies that the product (e.g., meat) is more tender and requires less force when slicing or chewing. From a food quality perspective, products with this characteristic are often preferred for their improved taste profile (Barido et al., 2020). Accurate determination of livestock and poultry amino acid requirements can improve the efficiency of protein utilization by animals, thus reducing feed costs and increasing production efficiency (Awawdeh, 2022).

In recent years, limited supplementation of protein feed with the key restricted amino acids (methionine and lysine) and the high cost of breeding have hindered development of the feed industry and animal husbandry. One approach to solving these issues is the need to develop new protein feed resources. Currently, the production of methionine is undertaken predominantly by chemical synthesis. However, chemically synthesized products have several disadvantages including poor safety standards, serious environmental pollution and high costs. Recently, the biosynthesis of methionine via microbial fermentation has gained growing attention owing to certain advantages including less environmental damage and low cost of raw materials. With the continuous growth of the biotechnology field, new microbial strains are routinely being developed to construct microbial cell factories for the production of methionine. Zein (*δ-zein*) is a protein rich in sulfur-containing amino acids (20% methionine) present in the endosperm of maize (Kim and Krishnan, 2003). Kim et al. increased the methionine content in transgenic plants by expressing *δ-zein* in soybean (Kim and Krishnan, 2019).

*C. utilis* is a yeast approved by the Food and Drug Administration (FDA) for use as a Generally Recognized as Safe (GRAS) food additive (Li et al., 2021). *C. utilis*, as a source of protein in concentrated feed for dairy cows, can be used as a substitute for soybean meal without affecting the quality of Norwegian Gouda-type cheeses (Olsen et al., 2021). Kieliszek et al. reported that *C. utilis* cells performed the biotransformation of inorganic selenium to organic derivatives (e.g., selenomethionine). It creates the possibility of obtaining selenium biocomplexes that can be used in the production of protein-selenium dietary supplements for animals and humans. *C. utilis* cellular biomass enriched with organic forms of selenium may be a protein source with high potential for application in the livestock and drug industries (Kieliszek et al., 2017). *C. utilis* has been used as a cell factory for protein expression of a wide range of recombinant products. The advantages of utilizing *C. utilis* include food safety and fast growth rates with an important role in the feed additives. However, limited studies on its genetic background and regulatory elements have been undertaken where this information will be key in improving the expression capacity of *C. utilis.*

As one of the basic elements for gene transcription, promoters play an important role in regulating gene expression and optimizing metabolic pathways (Nguyen et al., 2024). Constitutive promoters have a wide range of applications in metabolic engineering and synthetic biology. These types of promoters are independent of the host growth environment and growth stage and can maintain a relatively stable level of gene expression (Cui et al., 2016). The glyceraldehyde-3-phosphate dehydrogenase (*GAP*) promoter has been widely used as a constitutively strong promoter for high-level protein production. The strength of the *GAP* promoter is usually considered to be comparable to that of the methanol-induced *AOX1* promoter (Vogl, 2022). Error-prone PCR (EP-PCR) is an approach commonly used in synthetic biology and directed evolution for integrating mutations across a wide range of genetic backgrounds (Feng et al., 2018; Myers, 2023). The imperfect fidelity of DNA polymerase is exploited to mismatch non-complementary dinucleotides during template mediated extension, followed by insertion of a mutation on the newly synthesized daughter strand, which is then inherited by the double-stranded progeny during replication. The obtained random mutant sequences are cloned into vectors to construct mutant libraries, and then suitable methods are used to screen promoter mutants that drive improved target protein expression (Feng et al., 2018). Qin et al. constructed promoter libraries by EP-PCR to screen for promoters of different strengths (Qin et al., 2011a). Hanson et al. developed a detailed method to construct a library of cloned mutants through EP-PCR and used the online program PEDEL-AA to analyze the diversity in the EP-PCR randomized library (Firth and Patrick, 2008). The advantage of the EP-PCR generated random mutants library is that, in contrast to targeted mutagenesis and rational design, EP-PCR is a highly stochastic process, and directed evolution utilizes Darwinian principles to identify proteins with novel or improved properties (Hanson-Manful and Patrick, 2013). Zhang et al. obtained promoters with different abilities to induce expression by modifying the maltose-inducible promoter P*glvc* using EP-PCR. With green fluorescent protein expression as a readout, the study reported that the induced expression intensity from the promoter mutant was about 3.15-fold higher than that of the original promoter, proposing its potential application in metabolic engineering and synthetic biology (Zhang et al., 2022).

Signal peptides (SPs) are important regulatory elements that guide protein secretion. The majority of SPs are located at the N-terminus of secreted proteins with a small number found at the C-terminus or within the protein (Yiping Shi et al., 2014). SPs are a key factor that determines the optimal pathway of target proteins and how they are secreted across the cell membrane. The role of the SP is important to the production of soluble recombinant proteins, vaccine candidates and diagnostic proteins (Zhang et al., 2019). Different signal peptides can greatly affect the secretion levels of target proteins (Xue et al., 2023). The construction of signal peptide libraries in combination with high throughput screens enables identification of optimal signal peptides that drive different efficiencies of target protein secretion. This strategy has been used successfully to optimize the secretion efficiency of many proteins in heterologous hosts (Mori et al., 2015). In a recent study, Liu et al. identified a ferulic acid esterase with broad substrate specificity that is expressed and secreted by *Bacillus subtilis*. After codon optimization and signal peptide library screening, the secretion of feruloyl esterase by the strain was 10.2-fold higher than that of the parent strain. Hence, *B. subtilis* could improve secretion of heterologous enzymes by altering the protein sequences (Liu P. et al., 2022).

**Figure 1:**
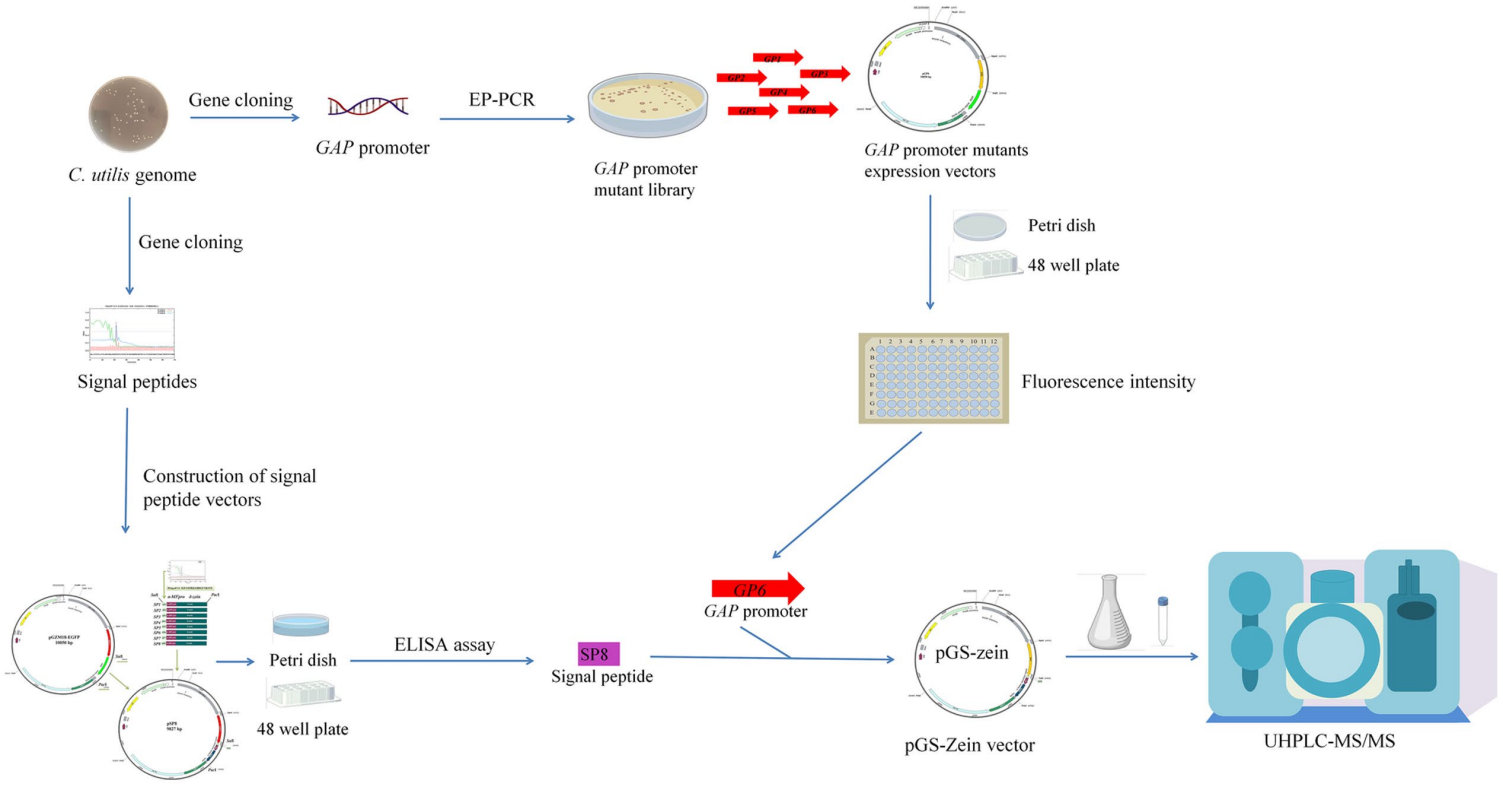
Schematic diagram of the overall strategy for enhancing methionine yields in an engineered *C. utilis*. A *C. utilis GAP* promoter mutant library was constructed and screened to obtain a strong promoter. The endogenous signal peptides from *C. utilis* were identified by sequencing the *C. utilis* genome. The strong promoter and an optimal signal peptide were utilized to enhance methionine production by overexpressing *δ-zein* gene in a recombinant *C. utilis.*

In this study, a *C. utilis GAP* promoter mutant library was constructed and screened to obtain a strong promoter. Subsequently, eight endogenous signal peptides from *C. utilis* were identified by sequencing the *C. utilis* genome. Finally, the strong promoter GP6 and an optimal signal peptide SP8 were utilized to enhance methionine production by overexpressing the *δ-zein* gene in a recombinant *C. utilis.* A schematic diagram of the overall strategy for enhancing methionine yields in an engineered *C. utilis* is shown in Figure 1.

## 2 Materials and methods

### 2.1 Construction of the reporter gene vector pGZM18-EGFP

All primer sequences used for strain construction and identification are listed in Supplementary Table S1. All plasmids and strains used in this study are listed in Supplementary Tables S2, S3. *Escherichia coli* cells were used for plasmid construction and cultured at 37°C in Luria Bertani (LB) medium. *C. utilis* cells were used as the host strain for target gene expression and cultivated at 28–30°C in Yeast Extract Peptone Dextrose (YPD) medium. If necessary, recombinant *C. utilis* YPD medium was supplemented with cycloheximide (CHX, 20 μg/ml).

To characterize the strengths of the *C. utilis* mutant promoters, a reporter gene vector pGZM18-EGFP was constructed using the expression vector pGZM18 and a reporter gene with enhanced green fluorescent protein (*egfp*). Plasmid pGZM18 carrying *δ-zein* gene was engineered in our previous work and deposited in our lab (He et al., 2018). The plasmid pGZM18 was used as a template to design primers for seamless cloning experiments, PCR amplification of the 18S rDNA gene fragment, *GAP* promoter, *GAP* terminator, and Large ribosomal subunit protein (*mL41*) gene. The *egfp* gene and *SV40 poly (A) signal* gene sequence were amplified by PCR from pCMV-C-EGFP plasmid (Beyotime, Shanghai, China). The pBR322 vector was subjected to *Eco*RV and *Nru*I double digestion, and the product was recovered by gel electrophoresis. The 18S rDNA gene fragment, *GAP* promoter, *egfp*, *GAP* terminator, and *mL41* gene were ligated with the linearized pBR322 vector at a particular molar ratio, gently mixed, and incubated at 50°C for 15 min. The recombinant products were placed on ice and cooled for transformation into *E. coli.* To construct the reporter strain C/pGP, plasmid pGZM18-EGFP was digested by *Nco*I and transformed into *C. utilis. C. utilis* competent cells were prepared according to the Yeastmaker™ Yeast Transformation System 2 User Manual (Takara, Beijing, Japan). Finally, the control vector with the original *GAP* promoter driven *egfp* gene expression was constructed as a reference.

### 2.2 Construction of recombinant *C. utilis* with *GAP* promoter mutants

The *GAP* promoter was mutated by error-prone PCR (EP-PCR). For the construction of the *C. utilis GAP* promoter mutants, the *GAP* promoter was amplified with primers QDZ-P2S and QDZ-P2AS (Supplementary Table S1) based on the reporter gene vector pGZM18-EGFP. EP-PCR was performed according to Diversify PCR Random Mutagenesis Kit standard protocols (Takara, Beijing, Japan). To increase mutational diversity and reach an appropriate error rate, four consecutive EP-PCRs were conducted. Directly ligating the obtained EP-PCR products into the *C. utilis* homologous integration expression vectors was relatively inefficient and resulted in only a small number of promoter mutants. Therefore, the EP-PCR products were first ligated into the pEASY-T1simple cloning vector and transformed into *E. coli*. After sequencing the promoter mutant DNA sequences, six promoter mutant sequences with the highest mutation rate were selected to screen and characterize strong promoters. Finally, six candidate promoter mutant sequences were inserted into pGZM18-EGFP cleaved with *Kpn*I and *Sal*I, respectively. Six *GAP* promoter mutant expression vectors were constructed and transformed individually into *E. coli.* The six expression plasmids were digested with *Nco*I and purified cleavage products were transformed into *C. utilis* to construct the *GAP* promoter mutant recombinant strains. Six *GAP* promoter mutant *C. utilis* strains were selected for further screening and characterization of strong *GAP* promoters.

### 2.3 Screening of *GAP* strong promoter

The *GAP* promoter mutant *C. utilis* strains that harbored *egfp* gene were screened based on the fluorescence signal. The *GAP* promoter mutant single colonies were selected and cultured into 1 ml YPD medium for 24 h. From each well of the YPD culture plates, 100 μl culture broth was taken and added to 900 μl of Buffered Minimal Dextrose (BMD, 0.2%) medium for 48 h. Then the 900 μl BMD medium (with 1% glucose) was cultured with a 100 μl culture from the BMD (0.2% glucose) plate and grown for 36 h (Qin et al., 2011b). The *GAP* promoter mutant *C. utilis* strains were cultured in 48 deep-well plates at 30°C, 220 r/min. The *egfp* gene expression intensity and strain concentration was measured by a multifunctional enzyme labeling instrument (BioTek, Vermont, USA). The culture broth was diluted with phosphate buffered saline (PBS) to a final OD_600nm_ of around 1 and 200 μl of the diluted sample was transferred to a 96-well plate. Fluorescence intensity was measured under excitation light at 488 nm and emission light at 550 nm, and the OD_600 nm_ was read.

The *GAP* promoter mutant single colonies were selected and cultured into 1 ml YPD medium for 48 h. The *egfp* gene transcript levels in different *GAP* promoter mutants were detected by quantitative real-time PCR (qRT-PCR). Total RNA samples were extracted from the cells using a total RNA extraction reagent (Takara, Beijing, Japan). Takara’s PrimeScriptTM RT Reagent Kit with gDNA Eraser was used to convert mRNA into cDNA by reverse transcription, which was used as a template for qRT-PCR. The qRT-PCR was performed according to Takara’s SYBR Premix Ex TaqTM instructions, and the relative transcript levels of *egfp* gene were analyzed by the 2^-ΔΔCt^ method using the *gapdh* gene as an internal reference (Zhang et al., 2021; Guan et al., 2016). The relative activities of six *GAP* promoter mutants were characterized by comparison with those of the original *GAP* promoter.

### 2.4 Construction of recombinant *C. utilis* with different endogenous signal peptide vector

To analyze the usability of signal peptide vectors for recombinant protein production in *C. utilis*, the *GAP* promoter was selected to drive the production of *δ-zein*. To identify the target proteins, we added a His-tag to the C-terminus of *δ-zein*. The *C. utilis* whole genome sequence was analyzed using SignalP 5.0 (Sanaboyana and Elcock, 2024) to obtain endogenous signal peptide sequences with protein secretion potential. The *C. utilis* amino acid sequences were predicted and analyzed by entering them into the SignalP 5.0 software based on the C, Y, S and D scores assigned by the software (Almagro Armenteros et al., 2019). The C score gives an idea about the role of different amino acids in the shear site. Y score reflects the combined highest point of S and C scores. The S score indicates contribution of different amino acids in the signal peptide region. D score is the average of S and Y scores, which is important to distinguish if a protein is secreted (Yiping Shi et al., 2014). Amino acid sequences with a D score of more than 0.7 have a high likelihood of being a signal peptide. Therefore, 8 signal peptide sequences were screened from the genome data. *Saccharomyces cerevisiae* (*S. cerevisiae*) *α*-mate-factor (a-MF) (Dai et al., 2024), as a widely used exogenous signal peptide with good secretion ability, was used as a signal peptide screening control vector to investigate the effect of *C. utilis* endogenous signal peptide on *δ-zein* secretion. A construct containing the *S. cerevisiae α*-MF signal peptide and *δ-zein* (C-terminal with His-tag) gene was synthesized (GenScript, Piscataway, USA). The synthesized construct was ligated to *Sal*I, *Pac*I double digested pGZM18-EGFP and pM-zein (C-terminal containing His-tag) vector was constructed as the experimental control vector for screening signal peptide sequences. α-MF signal peptide includes a pre-region and a pro-region. The α-MF pro-region was retained when the subsequent screening signal peptide vectors were constructed in the present study.

*C. utilis* genomic DNA was extracted by TIANamp Yeast DNA Kit (TIANGEN, Beijing, China) and was used as the template to amplify the signal peptides with the SP1-8F/SP1-8MR primers (Supplementary Table S1). α-MFpro-zein sequences were PCR amplified from pM-zein vector using the primers SP1-8MF/SPR. The amplified signal peptides and α-MFpro-zein sequences were used as nested PCR templates, and SP1-8F/SPR were used as primers to amplify eight different endogenous signal peptide sequences, respectively. The PCR products were digested with *Sal*I and *Pac*I, and cloned into pGZM18-EGFP that had been digested with the same enzymes to yield eight endogenous signal peptide expression vectors: pSP1, pSP2, pSP3, pSP4, pSP5, pSP6, pSP7 and pSP8. These vectors were transformed into *E. coli*. The signal peptide expression vectors as well as pM-zein were subsequently transformed into *C. utilis*, respectively.

### 2.5 Screening for optimized endogenous signal peptides

The content of His-tagged *δ-zein* protein in the supernatant of the fermentation broth of signal peptide recombinant *C. utilis* cultured for 48 h was detected using the enzyme linked immunosorbent assay (ELISA). The His-Tag ELISA assay kit was used in accordance with the product’s instruction manual (GenScript, Piscataway, USA). Recombinant protein content in the samples was determined by spectrophotometry (OD_450nm_). All experiments were performed in triplicates. The data were analyzed using SPSS22.0 statistical software. Analysis of variance (ANOVA) was used to determine significant differences among the results (means ± standard deviation).

### 2.6 Construction of engineered *C. utilis* with a strong promoter and optimized signal peptide

The vector pSP8 was digested with *Kpn*I and *Sal*I, and the *GAP* promoter mutant GP6 fragment was circularized using T4 DNA ligase to generate the constitutive vector. The *δ-zein* secretion expression vector containing the *GAP* promoter mutant GP6 and SP8 signal peptide sequence was constructed and the vector was subsequently transformed into *E. coli*. The vector bacterial DNA sequences from prokaryotic microorganisms, including the resistance screening marker Amp^r^, and prokaryotic replication regions, were later deleted by splicing overlap extension-PCR (SOE-PCR) to obtain a food-grade vector pGS-Zein, and the food-grade vector was transformed into *C. utilis*.

### 2.7 Ultra-high performance liquid chromatography coupled to tandem mass spectrometry (UHPLC–MS/MS) system analysis

Sample preparation was performed according to the following steps. The *C.utilis* culture solution was vortexed to ensure homogeneity. Ten microliters of the vortexed solution were then transferred into a microcentrifuge tube containing 490 μl of MS grade water. After vortexing to mix, 50 μl of the diluted sample was added to 200 μl of precipitation reagent containing mixed internal standards (acetonitrile: methanol = 1:1). After vortexing, the samples were incubated on ice for 30 min, centrifuged at 12,000 rpm for 10 min at 4°C, and the supernatants were used for UHPLC–MS/MS analysis.

An ultra-high performance liquid chromatography coupled to tandem mass spectrometry (UHPLC–MS/MS) system (ExionLC™ AD UHPLC-QTRAP 6500^+,^ Boston, USA) was used to quantitate the production of methionine with different promoters in *C. utilis* harboring the *δ-zein* gene. Separation was performed on an ACQUITY UPLC BEH Amide column (2.1 × 100 mm, 1.7 μm) which was maintained at 50°C. The mobile phase, consisting of 0.1% formic acid in 5 mM Ammonium acetate (solvent A) and 0.1% formic acid in acetonitrile (solvent B), was delivered at a flow rate of 0.30 mL/min. The solvent gradient was set as follows: initial 80% B, 0.5 min; 80–70% B, 2 min; 70–45% B, 4 min; 45–80% B, 6.01 min; 80% B, 9 min. The methionine content was determined in the three groups of samples: wild-type *C. utilis* (WT), C/pSP and C/pGS-zein. WT was used as control group 1, C/pSP engineered *C. utilis* containing original *GAP* promoter and *δ-zein* gene was used as control group 2, and optimized C/pGS-zein engineered *C. utilis* containing the *δ-zein* gene, *GAP* promoter mutant GP6, and signal peptide SP8 were used as the experimental group. The UHPLC–MS/MS system analysis was performed by Beijing Novozymes Technology Co. (China).

## 3 Results

### 3.1 Construction of GAP promoter mutants

**Figure 2:**
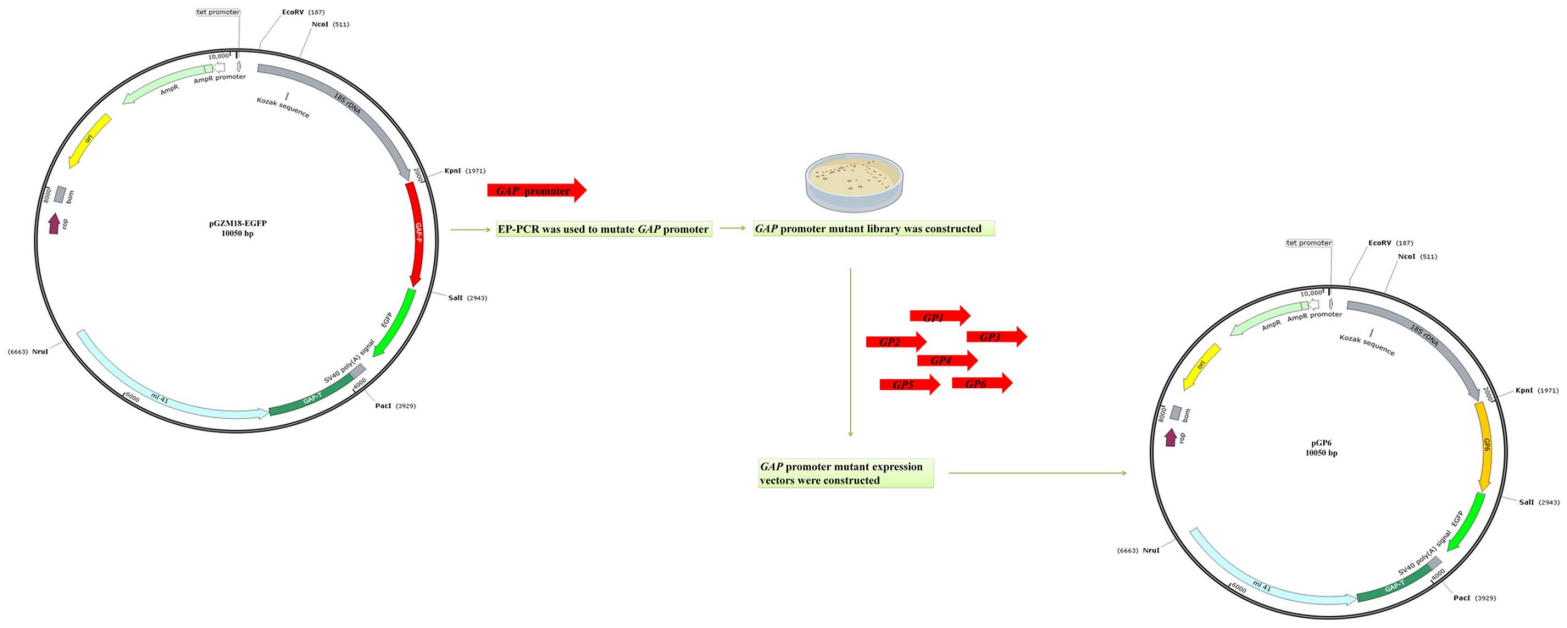
Schematic diagram of *GAP* promoter mutant expression vectors construction. A *GAP* promoter mutant library was generated by mutated *GAP* promoter variants obtained through EP-PCR. The six promoter mutant sequences with the highest mutation rate were selected for construction of recombinant *C. utilis* with *GAP* promoter mutants.

The reporter gene vector pGZM18-EGFP was constructed and used to test the expression of the *egfp* gene under the control of the original *GAP* promoter in the recombinant strain C/pGP. A *GAP* promoter mutant library was generated by mutated *GAP* promoter variants obtained through four consecutive rounds of EP-PCR (Figure 2). Forty putative promoter mutants were sequenced following EP-PCR. The sequencing results demonstrated that the mutation rate ranged from 0.3 to 1.9%, and six promoter mutant sequences with the highest mutation rate were selected for construction of recombinant *C. utilis* with *GAP* promoter mutants. It has previously been reported that promoter sequences with mutation rates ranging from 1 to 5% are generally effective promoters. Six sequences with the highest mutation rates (1.2 to 1.9%) were selected, and subsequent screening was carried out. The constructed *GAP* promoter mutants were introduced into the *C. utilis* chromosome by homologous recombination to obtain the *GAP* promoter mutant *C. utilis*. The *GAP* promoter mutant recombinant vectors were digested and identified. The full length of the plasmid was 10,050 bp after *Sal*I single digestion and *Kpn*I+*Sal*I double digestion resulted in a 970 bp fragment of the *GAP* promoter mutant and 9,080 bp for the remaining sequence of the vector. After single enzyme digestion of pGZM18-EGFP plasmid with *Sal*I, a linear fragment of 10,050 bp was obtained. After double enzyme digestion with *Kpn*I+*Sal*I, the length of original *GAP* promoter was 970 bp, and the remaining sequence length of *GAP* promoter was 9,080 bp. The results showed that the *GAP* promoter mutants expression vectors were successfully constructed (Supplementary Figure S1).

### 3.2 Characterization of different promoter mutants

Gene expression under the control of six selected *GAP* promoter mutants was further investigated at the transcriptional level. The *GAP* promoter mutants recombinant *C. utilis* were cultured and promoter activity was represented by the fluorescence intensity of *egfp* gene reporter. A significant increase in fluorescence intensity of the *GAP* mutant recombinant *C. utilis* strains was observed compared to *C. utilis* with the original *GAP* promoter. The relative fluorescence intensities of three mutant recombinant *C. utilis* strains were significantly different from the original *GAP* promoter in the recombinant strain C/pGP (Table 1). The relative promoter activities were normalized to the activity obtained with the original *GAP* promoter. The relative fluorescence intensity of C/pGP6 was 1.87-fold higher than that of the control C/pGP and had the highest fluorescence intensity value, while the relative fluorescence intensities of C/pGP3 and C/pGP5 were 1.23-fold and 1.26-fold higher than those of the control C/pGP.

**Table 1:** Fluorescence intensity value of recombinant *C. utilis* with *GAP* promoter mutants.

Strain	RFU/OD_600_	Relative fluorescence intensity^1^
C/pGP	2.32 ± 0.07	1
C/pGP1	2.47 ± 0.01	1.06 ± 0.03
C/pGP2	2.42 ± 0.003	1.04 ± 0.03
C/pGP3	2.86 ± 0.03	1.23 ± 0.02^*^
C/pGP4	2.29 ± 0.05	0.99 ± 0.05
C/pGP5	2.92 ± 0.07	1.26 ± 0.06^*^
C/pGP6	4.35 ± 0.04	1.87 ± 0.07^*^

1 Compared with the relative fluorescence intensity of C/pGP, *: *p* ≤ 0.05 (One-Way ANOVA).

**Figure 3:**
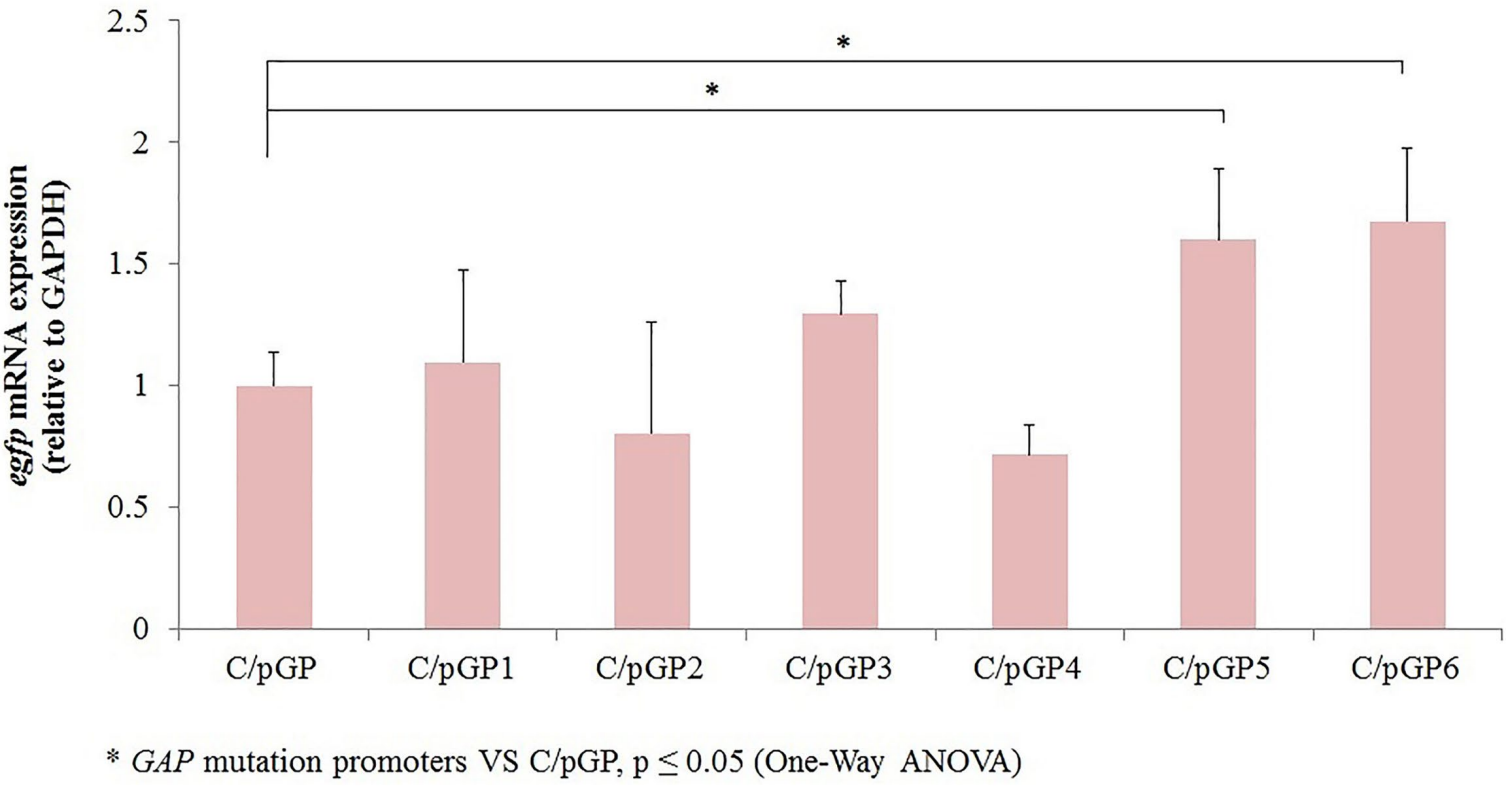
The relative transcription and protein expression level of recombinant *C. utilis* with GAP promoter mutants were determined by qRT-PCR. Detection of *egfp* transcription levels in recombinant* *C. utilis* with different *GAP* mutation promoters VS C/pGP, *p* ≤ 0.05 (One-Way ANOVA).

To detect the level of expression from different promoter mutants, transcript levels of the *egfp* gene were detected by qRT-PCR using the 2^-ΔΔCt^ method with the *gapdh* gene as an internal reference. Transcript levels of reporters were normalized to the value obtained with the original *GAP* promoter. The original promoter values were set to 1. The results showed that the relative expression of *egfp* mRNA in C/pGP3, C/pGP5 and C/pGP6 was higher than that in C/pGP, at 1.29-fold, 1.60-fold and 1.67-fold, respectively. The relative expression of *egfp* mRNA in C/pGP5 and C/pGP6 groups was significantly different from that in the C/pGP group (*p* ≤ 0.05). The relative *egfp* expression of C/pGP1, C/pGP2 and C/pGP4 was low, while the relative *egfp* expression of C/pGP6 was the highest. The trends of the qRT-PCR results and the fluorescence intensity results aligned well, both of which showed that GP6 had the strongest ability to induce expression (Figure 3).

### 3.3 Construction and characterization of different endogenous signal peptide vectors

**Figure 4:**
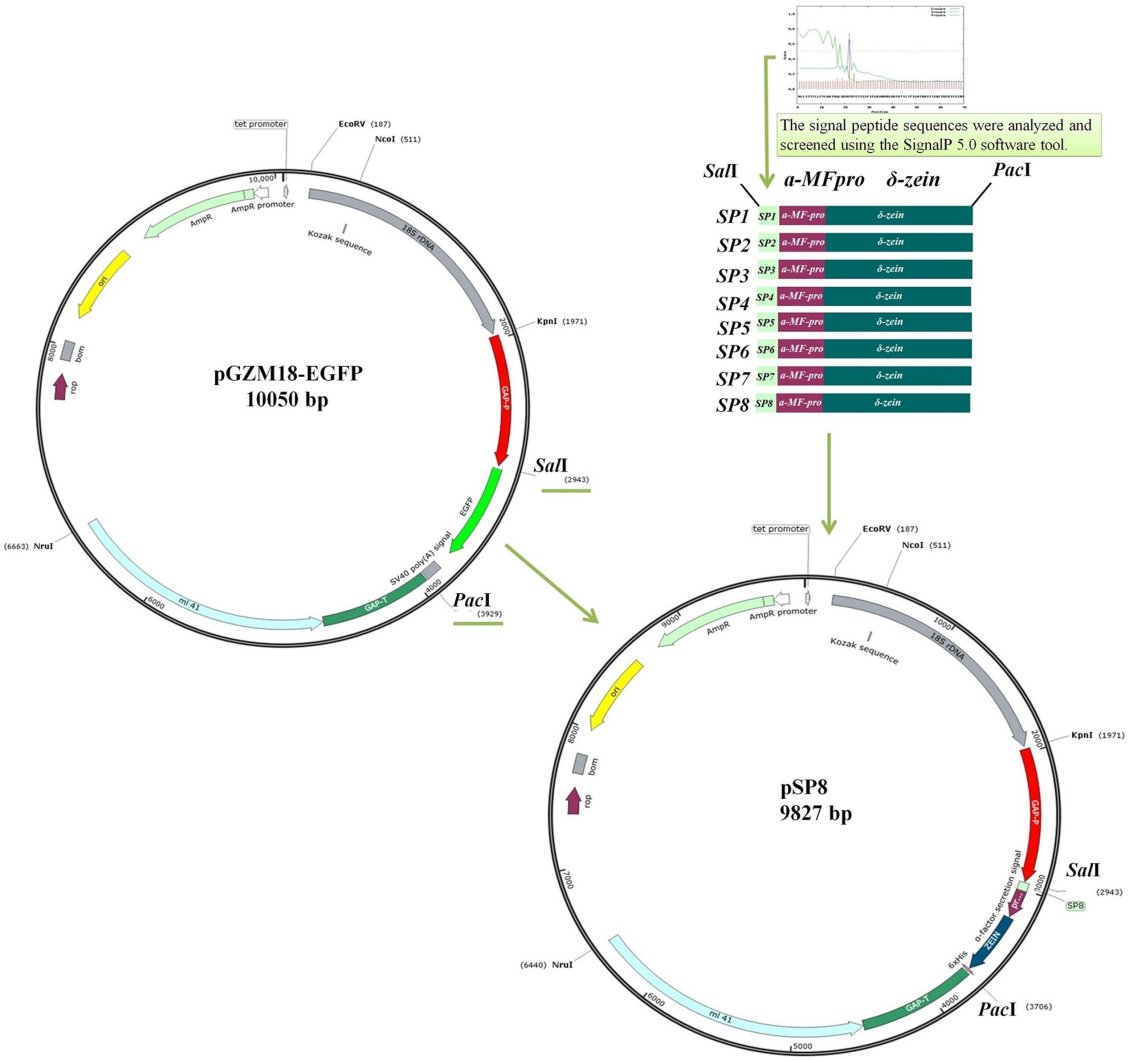
Schematic diagram of different endogenous signal peptide expression vectors construction.

Signal peptides are important factors that can affect protein secretion and expression. The endogenous signal peptides of *C. utilis* were analyzed by sequencing the *C. utilis* genome. Candidate signal peptides SP1, SP2, SP3, SP4, SP5, SP6, SP7, and SP8 were analyzed and the respective D scores were all greater than 0.8 (Table 2), indicating that all of the signal peptide sequences were functional signal peptides. The selected signal peptide sequences were amplified from the *C. utilis* genome. The *α*-MFpro-zein sequences were amplified from pM-zein. The SPs and α-MFpro-zein were used as nested PCR templates, and SP1-8F/SPR (Supplementary Table S1) was used as primer to amplify eight sequences of SP1-SP8α-MFpro-zein (C-terminal with His-tag). The amplified signal peptide sequences were digested with *Sal*I and *Pac*I and the target gene of 764 bp was recovered. pGZM18-EGFP was digested with *Sal*I+*Pac*I and the vector fragment of 9,060 bp was recovered by gel electrophoresis. Different signal peptide expression vectors were constructed (Figure 4) and named pSP1, pSP2, pSP3, pSP4, pSP5, pSP6, pSP7 and pSP8 (Supplementary Table S2). These vectors were transformed into *C. utilis* and the efficiency of each signal peptide were indicated by the His-Tag ELISA assay kit to evaluate secretory efficiencies of signal peptides.

**Table 2:** *C. utilis* genome signal peptide analysis.

Signal Peptide	Signal Peptide sequence	D Score	Cleavage site
SP5	MHLFFLLLALFFSPSVRA/ASESFALELDTD	0.941	AA18-19
SP3	MIPCTLLFLGLLASTVSA/AETCNSTQSCPE	0.917	AA18-19
SP2	MVAMMKFTLLLALGSLVSA/GFYDKSPVMEL	0.916	AA19-20
SP1	MHLFNSLACILLSLTTFTCA/KAPENHNLEK	0.895	AA20-21
SP4	MLNIFTLTLALMATTTLA/FDASAKDNMVLY	0.887	AA18-19
SP7	MKFLTSVLSTFLLAASCVCA/KEVDGVFTSV	0.873	AA20-21
SP8	MKFLTSVLSTFLLAASYVCA/KEVDGVFTSV	0.863	AA20-21
SP6	MLPIAVLSILGLATSVQA/TISCSSSSQCPE	0.837	AA18-19

**Figure 5:**
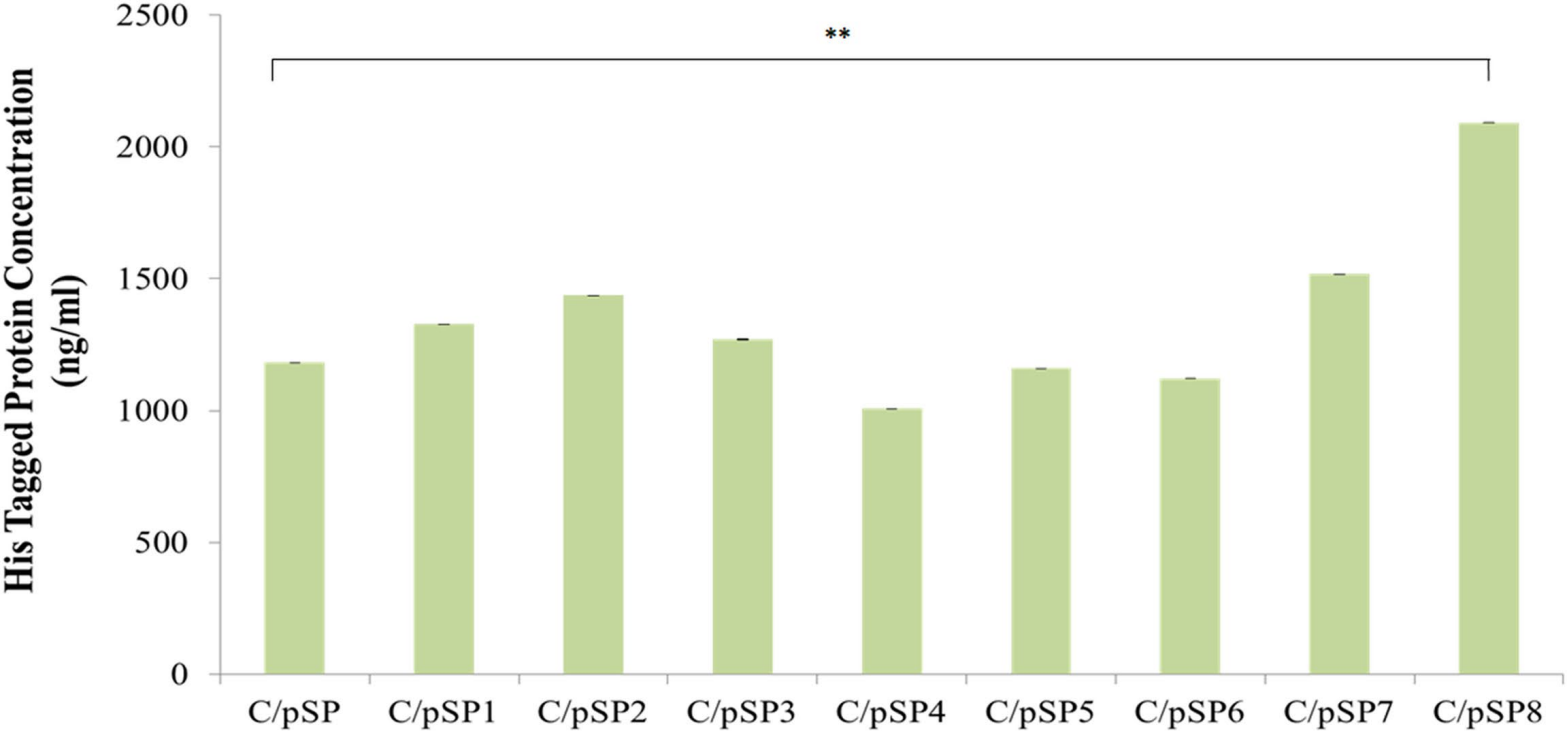
The effects of different signal peptides on *δ-zein* secretion were detected by ELISA. **: *p* ≤ 0.01 (One-Way ANOVA).

The content of His-tagged *δ-zein* fusion protein in the recombinant *C. utilis* supernatants was quantified. The content of His fusion *δ-zein* in the supernatant of C/pSP, C/pSP1, C/pSP2, C/pSP3, C/pSP4, C/pSP5, C/pSP6, C/pSP7 and C/pSP8 was 1181.70, 1326.87, 1436.52, 1270.40, 1007.00, 1159.97, 1122.09, 1517.57 and 2091.58 ng/mL, respectively. Protein content in C/pSP8 was significantly higher compared with the control group C/pSP (a-MF) (relative to the C/pSP group, *p* ≤ 0.01). Thus, signal peptide SP8 was selected as the optimal signal peptide to be used in the subsequent construction of optimized expression vectors (Figure 5).

### 3.4 The effect of strong promoter and optimized signal peptide on *C. utilis* expression

The optimized methionine-producing food grade engineered *C. utilis* were obtained by constructing expression vectors with the screened strong promoter GP6 and the optimal signal peptide pSP8. The methionine content in the engineered *C. utilis* C/pGS-zein increased by 21.09% compared to C/pSP engineered *C. utilis* containing original GAP promoter. In addition, the level of methionine content in C/pGS-zein was 33.64% more than that of the wild-type strain (Table 3). The results indicate that the system compromised of plasmid pGS-zein with promoter GP6 and signal peptide SP8 has tremendous potential for the overproduction of methionine by engineered *C. utilis*. This approach can be adapted to improve the production of heterologous proteins in *C. utilis*.

**Table 3:** Detection of methionine content.

Sample group	Methionine content (μg/mL)
WT	38.01 ± 2.686
C/pSP	41.95 ± 5.755
C/pGS-zein	50.80 ± 3.183

## 4 Discussion

L-Methionine is a sulfur-containing amino acid essential for human and animal nutrition, mainly used in the feed industry and is an indispensable additive in animal feed (Gong et al., 2023). Currently, research on the production of methionine by microbial fermentation mainly focuses on the cultivation of suitable strains through classical mutagenesis (Cai et al., 2023). In recent years, the research direction has shifted towards metabolic engineering of major hosts such as *E. coli* (Niu et al., 2023), *Corynebacterium glutamicum* (*C. glutamicum*) (Liu B. et al., 2022) and *Bacillus cereus* (*B. cereus*) (He et al., 2020). A more specific and targeted approach to improve methionine production in engineered strains is to understand and take advantage of the regulatory mechanisms used by the host (Schipp et al., 2020). *C. utilis* has properties such as amino acid diversity and protein content of about 50% of the weight of the cells, making it an ideal source of protein supplementation for animal feed (Buerth et al., 2011). *C. utilis* is a Crabtree negative yeast, which does not produce ethanol under aerobic culture conditions, and has high respiratory capacity, which allows it to be cultured at high density under efficient continuous culture conditions (Li et al., 2022). These growth conditions are conducive to large-scale culturing, which improves industrial productivity and reduces costs, and *C. utilis* can be used in various fields such as feed additives and biopharmaceuticals (Kunigo et al., 2013). Currently, research on *C. utilis* expression systems and regulatory components is limited and the best host vector system has yet to be established. This requires the development of an optimized gene expression system that necessitates selection of appropriate strains, expression vectors, promoters, and signal peptides. In this study, the genome and sequence analysis of *C. utilis* was performed to obtain the sequence of endogenous signal peptides, construct *δ-zein* expression vectors with different signal peptides and their recombinant yeast strains and screen for the optimal signal peptide of *δ-zein*. In addition, we used EP-PCR to mutate the *C. utilis GAP* promoter and construct a promoter mutant library. Using EGFP as a reporter protein, recombinant yeast was constructed and screened for strong *GAP* promoter mutants.

Promoters serve as crucial regulatory components within the genome, overseeing the spatial and temporal expression of genes as well as the levels at which they are expressed (Liu et al., 2019). In particular, strong promoters are essential in biotechnological applications and associated industrial sectors. Qin et al. constructed a *GAP* promoter library by mutagenesis and obtained *GAP* promoter mutants with activities ranging from 0.006 to 19.6 times the activity of the original GAP promoter (Qin et al., 2011a). Ata et al. constructed a library of *GAP* promoters with different regulatory properties and overexpressed or deleted selected transcription factor genes to understand their role in heterologous protein production. Overexpression in the range of 0.35 to 3.10-fold higher than the original *GAP* promoter was reported (Ata et al., 2017).

The SP plays a vital role in the successful directing of the translocation machinery and the subsequent movement from the cytoplasm into the culture medium. At present, research on the *C. utilis* endogenous signal peptide is limited. Therefore, further research on *C. utilis* is necessary to gain an in-depth understanding of the interaction between signal peptides and proteins to improve the secretion and expression efficiency of the target protein. This has significant consequences for future research and application of *C. utilis* in the field of biotechnology. Consequently, the effective extracellular production of target proteins would greatly benefit from a suitable SP. Mayer et al. created a library of signal peptides from *Priestia megaterium* (*P. megaterium*) to develop a simple and rapid cloning and screening system to identify signal peptides that are optimal for different proteins. The results showed a 1.6-fold increase in the secretion of the *α*-amylase AmyE, demonstrating the functionality of the entire library and suggesting that this approach contributes to the improved secretion of recombinant proteins from Bacillus (Mayer et al., 2022).

Most research on methionine production via microbial fermentation has hitherto focused on traditional mutagenesis or metabolic engineering of well-characterized hosts such as *E. coli* and *C. glutamicum*. Through analyzing the rate-limiting factors in the L-methionine synthesis pathway, Li optimized the biosynthetic pathway, significantly boosting methionine production in *E. coli* to 10 g/L via fermentation (Li, 2017). Gao performed metabolic engineering modifications using *C. glutamicum* as the parental strain, yielding two engineered strains with methionine production levels of 3.78 g/L and 4.32 g/L, 2.96-fold and 2.57-fold higher than the wild-type strain, respectively (Gao, 2022). It is worth noting that, compared with our study, these studies adopted different metabolic engineering modification methods and achieved very high methionine yields. In future research, we will optimize the fermentation processes of engineered strains and conduct metabolic engineering modifications targeting methionine synthesis-related pathways to further increase methionine yields. *C. utilis*, a type of single-cell protein (SCP), offers advantages such as a short production cycle, straightforward manufacturing process, high nutritional value, and low production cost (Krahulec et al., 2020). A significant hurdle in utilizing *C. utilis* for recombinant protein production and enhanced methionine synthesis is its poorly understood genetic background. This study is the first to construct a GAP promoter mutant library and analyze endogenous signal peptides by sequencing, thereby uncovering new *C. utilis* genetic resources. This expands the genetic toolkit for this yeast species while providing a foundation for further genetic engineering efforts, particularly for *C. utilis*-based methionine production. The overall strategy of improving methionine production in *C. utilis* through promoter and signal peptide optimization aligns with the growing demand for sustainable and cost effective production methods. Chemically synthesized methionine has drawbacks such as safety concerns, environmental pollution, and high costs. Microbial fermentation, especially using a food-grade organism like *C. utilis*, offers a more sustainable alternative. By optimizing genetic elements in *C. utilis*, this study paves the way for large-scale, efficient, and environmentally friendly methionine production. This approach can be extended to other heterologous protein production applications utilizing *C. utilis*, thereby contributing to the development of a more sustainable biotechnology industry.

## 5 Conclusion

The *GAP* promoter of *C. utilis* was studied for the first time. We constructed a mutant library of the *GAP* promoter in *C. utilis*, screened and obtained a strong promoter, GP6. The genome of *C. utilis* was sequenced and endogenous signal peptides were analyzed. Homologous integrated expression vectors of different signal peptides and their recombinant *C. utilis* were constructed. The signal peptide SP8 enabled the highest expression and secretion of *δ-zein*. The combination of the strong promoter GP6 and the optimal signal peptide SP8 resulted in the construction of an integrated expression vector and its food-grade engineered *C. utilis* C/pGS-zein. Methionine content in the engineered *C. utilis* C/pGS-zein increased by 21.09% compared with engineered *C. utilis* C/pSP containing the original *GAP* promoter and 33.64% compared to wild-type *C. utilis*. This strategy of identifying a strong promoter and optimal signal peptide acts as an effective method for enhanced production of methionine in *C. utilis*. Further modifications via metabolic engineering of methionine synthesis related pathways can be undertaken to improve methionine yield in engineered *C. utilis*.

## Data Availability

The datasets presented in this study can be found in online repositories. The names of the repository/repositories and accession number(s) can be found in the article/Supplementary material.
