# Supplementary material for: An optimised promoter and signal peptide improves methionine production of a genetically engineered Candida utilis harboring the δ-zein gene
Source: Front Microbiol. 2025 Jul 4;16:1586229. doi: 10.3389/fmicb.2025.1586229 (PMC12271212; doi:10.3389/fmicb.2025.1586229)

Supplementary Material

An Optimised Promoter and Signal Peptide Improves Methionine Production of a Genetically Engineered *Candida utilis* Harboring the *δ-zein* gene

**Qiburi He^1^, Shaofeng Su^1^, Riqilang Ao^2^, Lingli He^3^, Xiao Wang^1^, Mei Chun^1^ and Gaowa Gong^1*^**

***Correspondence**: Gaowa Gong, gaowa2009c@163.com

# Supplementary Tables

**Table S1. Primers used in this study**

| Primer name | Primer sequence (5'-3') |
| --- | --- |
| 18s rDNAF | TGCCGGGCCTCTTGCGGGATGTAGTCATATGCTTGTCTCAAAGA |
| 18srDNAR | CGCCGGATCCTCCGCAGGTTCACCTACGG |
| GAPF | AACCTGCGGAGGATCCGGCGCGCCGGTAC |
| GAPR | TGCTCACCATGCATGCGCTAGCGTCGAC |
| EGFPF | TAGCGCATGCATGGTGAGCAAGGGCGAG |
| EGFPR | GTCATACAATTTAATTAATAAGATACATTGATGAG |
| GAP-TF | TATTAATTAAATTGTATGACTTTTATTTATGGGA |
| GAP-TR | GACCAAACCCACGTGTAATACCTCAGGAGT |
| CYHF | TATTACACGTGGGTTTGGTCTATGTTGCTGTG |
| CYHR | GGCCATCCAGCCTCGCGTCGAGTAAGTATGAAAAGAGCCAATGT |
| 2GAPS: | TGACGCTTCTTAGAGGGACT |
| 2GAPAS: | GGGCATGGCGGACTT |
| GFPS | TCTAGAATGGTGAGCAAGGGCGAG |
| GFPAS | GCTAGCTTAAGATACATTGATGAG |
| RTEGFPS | CGAGGGCGAGGGCGATG |
| RTEGFPAS | CCGTCGTCCTTGAAGAAGATGG |
| RTGAPDHS | GGTGCTGAGAAGGTTGACTACG |
| RTGAPDHAS | GGTGCTGAGAAGGTTGACTACG |
| SP1F | ACGCGTCGACATGCACTTGTTCAATAGCTTAGCG |
| SP1MF | CTTTAACCACTTTTACATGCGCTGCTCCAGTCAACACTACAACA |
| SP1MR | TGTTGTAGTGTTGACTGGAGCAGCGCATGTAAAAGTGGTTAAAG |
| SP2F | ACGCGTCGACATGGTAGCGATGATGAAGTTC |
| SP2MF | CTGGGATCACTGGTGAGTGCTGCTCCAGTCAACACTACAACA |
| SP2MR | TGTTGTAGTGTTGACTGGAGCAGCACTCACCAGTGATCCCAG |
| SP3F | ACGCGTCGACATGATCCCTTGTACACTGCTG |
| SP3MF | TTAGCCTCAACTGTGAGTGCCGCTCCAGTCAACACTACAACA |
| SP3MR | TGTTGTAGTGTTGACTGGAGCGGCACTCACAGTTGAGGCTAA |
| SP4F | ACGCGTCGACATGCTTAACATTTTCACGCTC |
| SP4MF | TGGCTACAACTACATTGGCCGCTCCAGTCAACACTACAACA |
| SP4MR | TGTTGTAGTGTTGACTGGAGCGGCCAATGTAGTTGTAGCCA |
| SP5F | ACGCGTCGACATGCATCTATTCTTTCTCTTG |
| SP5MF | CTTCTCACCTTCTGTCAGAGCAGCTCCAGTCAACACTACAACA |
| SP5MR | TGTTGTAGTGTTGACTGGAGCTGCTCTGACAGAAGGTGAGAAG |
| SP6F | ACGCGTCGACATGTTACCAATTGCAGTTCT |
| SP6MF | TCGCTACGAGTGTTCAGGCAGCTCCAGTCAACACTACAACA |
| SP6MR | TGTTGTAGTGTTGACTGGAGCTGCCTGAACACTCGTAGCGA |
| SP7F | ACGCGTCGACATGAAGTTTCTTACAAGTGTG |
| SP7MF | GCAGCATCATGTGTGTGTGCAGCTCCAGTCAACACTACAACA |
| SP7MR | TGTTGTAGTGTTGACTGGAGCTGCACACACACATGATGCTGC |
| SP8F | ACGCGTCGACATGAAGTTTCTCACAAGTGTAC |
| SP8MF | CGGCATCATATGTCTGCGCAGCTCCAGTCAACACTACAACA |
| SP8MR | TGTTGTAGTGTTGACTGGAGCTGCGCAGACATATGATGCCG |
| GAPF | CCTCTGTTTTCTCCTCATCAC |
| SPR | CCTTAATTAATCAATGATGATGATGATGATGGC |
| XHTS | TGGTTCCGCCCTCCCTGTCT |
| XHTAS | TGCCATAGAAGCGTTTAGCC |
| RTZS | AATGTTGGCTTTGTTTGCTTTGTTG |
| RTZAS | CAAGTGACCTGGAATGTGAGTAGC |
| RTGS | CTTGAGAGTTCCAACCACCGATG |
| RTGAS | CAGCCTTGACAGCAGCCTTG |
| GS1 | TGCCTGGAGCCGAGAAT |
| GS2 | CGTGCCATAGAAGCGTTTAG |

**Table S2. Plasmids used in this study**

| Plasmid name | Properties | Source |
| --- | --- | --- |
| pCMV-C-EGFP | harboring *egfp* gene | Beyotime company |
| pGZM18 | harboring *δ-zein* gene *GAP* promoter | our lab |
| pGZM18-EGFP | pGZM18 derivative vector，harboring *egfp* gene and *GAP* promoter | this study |
| pGP1 | pGZM18-EGFP derivative vector，harboring *GP1* promoter | this study |
| pGP2 | pGZM18-EGFP derivative vector，harboring *GP2* promoter | this study |
| pGP3 | pGZM18-EGFP derivative vector，harboring *GP3* promoter | this study |
| pGP4 | pGZM18-EGFP derivative vector，harboring *GP4* promoter | this study |
| pGP5 | pGZM18-EGFP derivative vector，harboring *GP5* promoter | this study |
| pGP6 | pGZM18-EGFP derivative vector，harboring *GP6* promoter | this study |
| pUC57-M-zein | harboring *δ-zein*，with α-MF signal peptide and His-tag | Synthesized by GenScript company |
| pM- zein | pGZM18-EGFP derivative vector，with α-MF signal peptide and His-tag | this study |
| pSP1 | pM-zein derivative vector，with SP1 signal peptide | this study |
| pSP2 | pM-zein derivative vector，with SP2 signal peptide | this study |
| pSP3 | pM-zein derivative vector，with SP3 signal peptide | this study |
| pSP4 | pM-zein derivative vector，with SP4 signal peptide | this study |
| pSP5 | pM-zein derivative vector，with SP5 signal peptide | this study |
| pSP6 | pM-zein derivative vector，with SP6 signal peptide | this study |
| pSP7 | pM-zein derivative vector，with SP7 signal peptide | this study |
| pSP8 | pM-zein derivative vector，with SP8 signal peptide | this study |
| pGS-zein | pSP8 derivative vector，harboring *GP6* promoter | this study |

**Table S3. Strains used in this study**

| Strain name | Properties | Source |
| --- | --- | --- |
| C/pGP | containing pGZM18-EGFP vector，with *egfp* gene and *GAP* promoter | this study |
| C/pGP1 | harboring *GP1* promoter，with *egfp* gene | this study |
| C/pGP2 | harboring GP2 promoter，*egfp* gene | this study |
| C/pGP3 | harboring GP3 promoter，*egfp* gene | this study |
| C/pGP4 | harboring GP4 promoter，*egfp* gene | this study |
| C/pGP5 | harboring GP5 promoter，*egfp* gene | this study |
| C/pGP6 | harboring GP6 promoter，*egfp* gene | this study |
| C/ pSP | containing pM-zein vector harboring *δ-zein* gene，with α-MF signal peptide and His-tag | this study |
| C/ pSP1 | harboring *δ-zein* gene，with SP1 signal peptide | this study |
| C/ Psp2 | harboring *δ-zein* gene，with SP2 signal peptide | this study |
| C/ pSP3 | harboring *δ-zein* gene，with SP3 signal peptide | this study |
| C/ pSP4 | harboring *δ-zein* gene，with SP4 signal peptide | this study |
| C/ pSP5 | harboring *δ-zein* gene，with SP5 signal peptide | this study |
| C/ pSP6 | harboring *δ-zein* gene，with SP6 signal peptide | this study |
| C/ pSP7 | harboring *δ-zein* gene，with SP7 signal peptide | this study |
| C/ pSP8 | harboring *δ-zein* gene，with SP8 signal peptide | this study |
| C/pGS-zein | harboring *δ-zein* gene，with GP6 promoter and SP8 signal peptide | this study |

Figure legend

Figure S1. The single and double enzyme digestion reaction verification of recombinant plasmid with GAP promoter mutant and pGZM18-EGFP plasmid. M1: Trans 2K Marker; 1,3,5,7,9,11,13: SalI single enzyme digestion reaction product; 2,4,6,8,10,12,14: KpnI, SalI enzyme digestion reaction product; M2: DL10000 DNA Marker.

**Supplementary Figure S1**


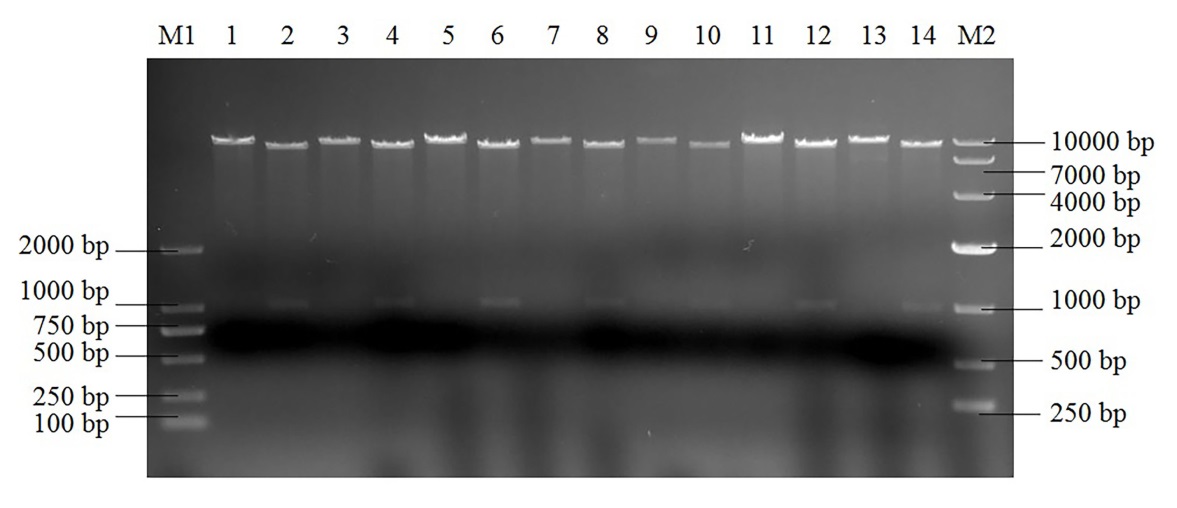


**Promoter sequences:**

GP


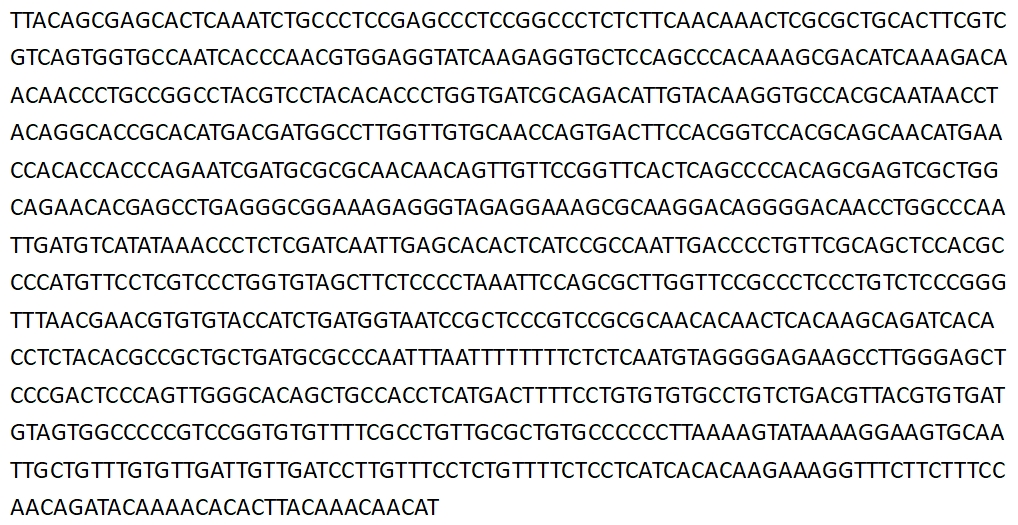


GP1


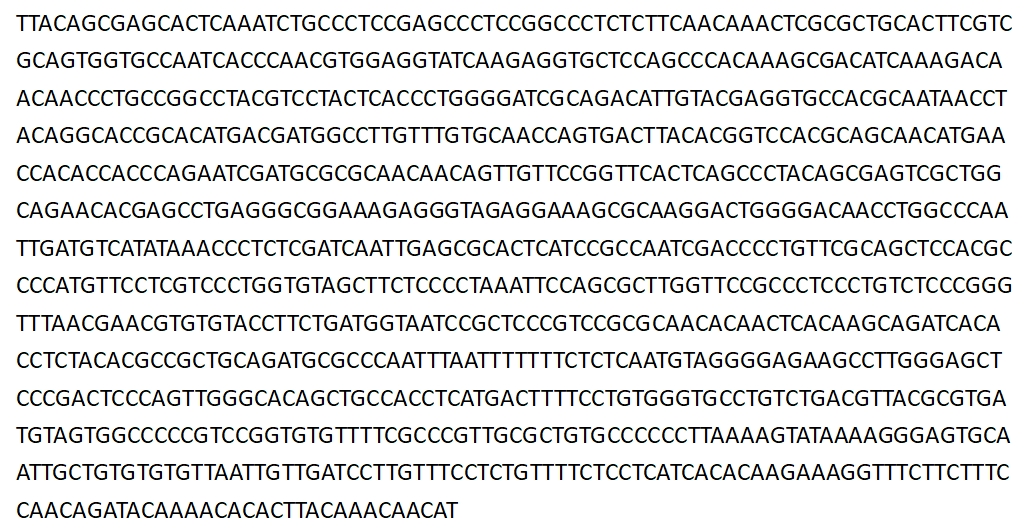


GP2


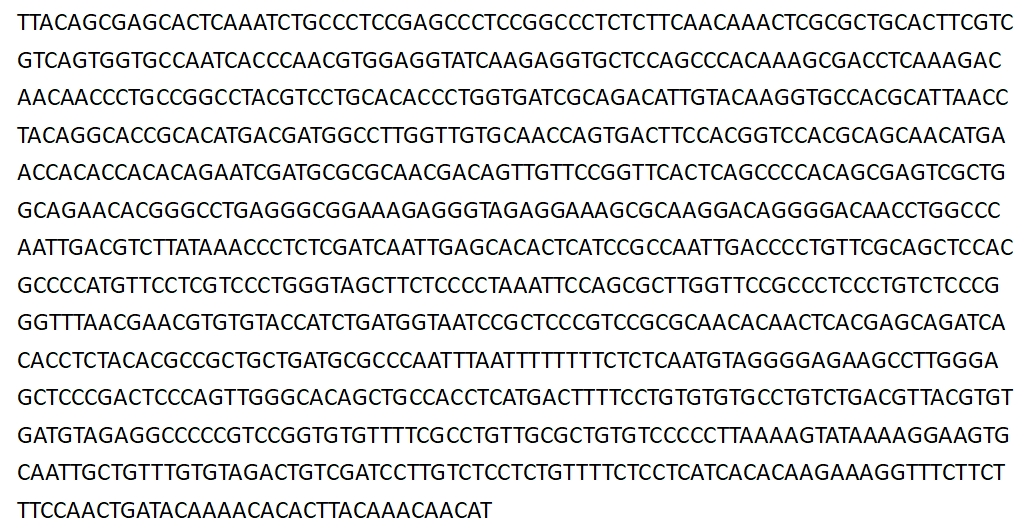


GP3


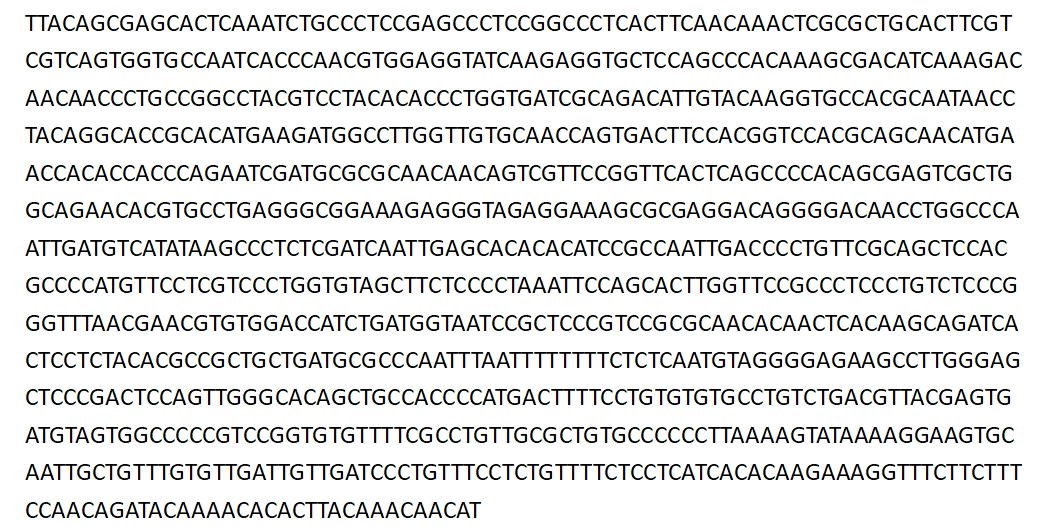


GP4


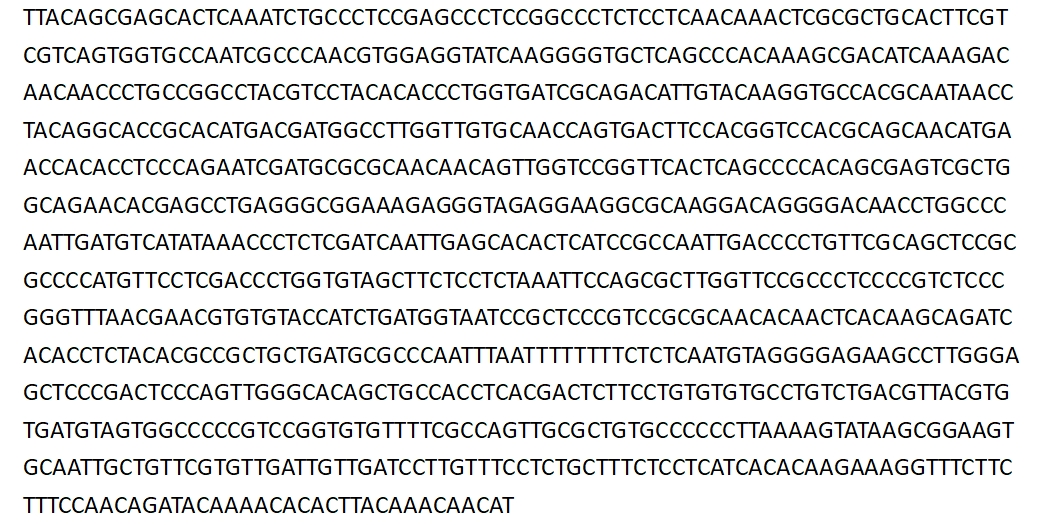


GP5


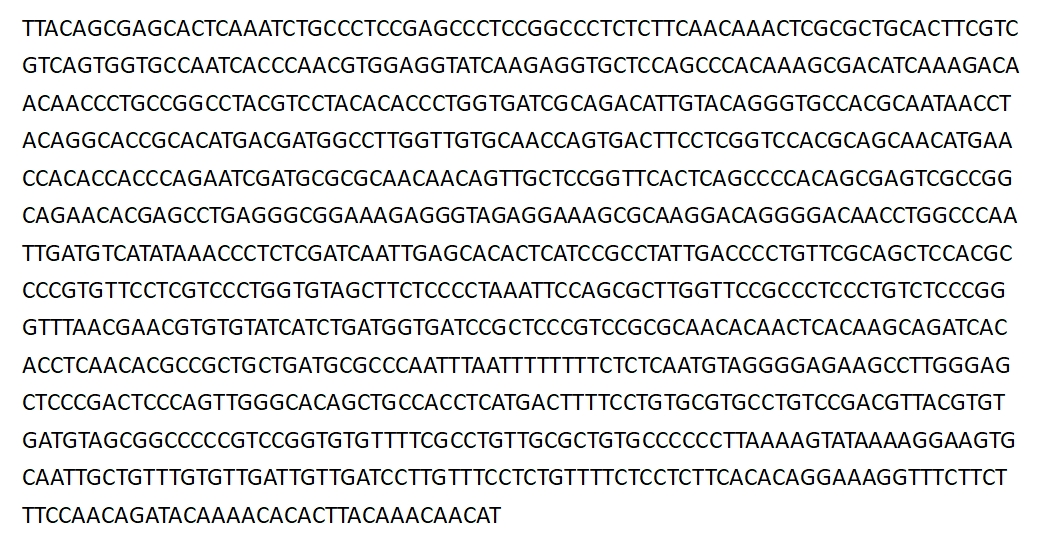


GP6


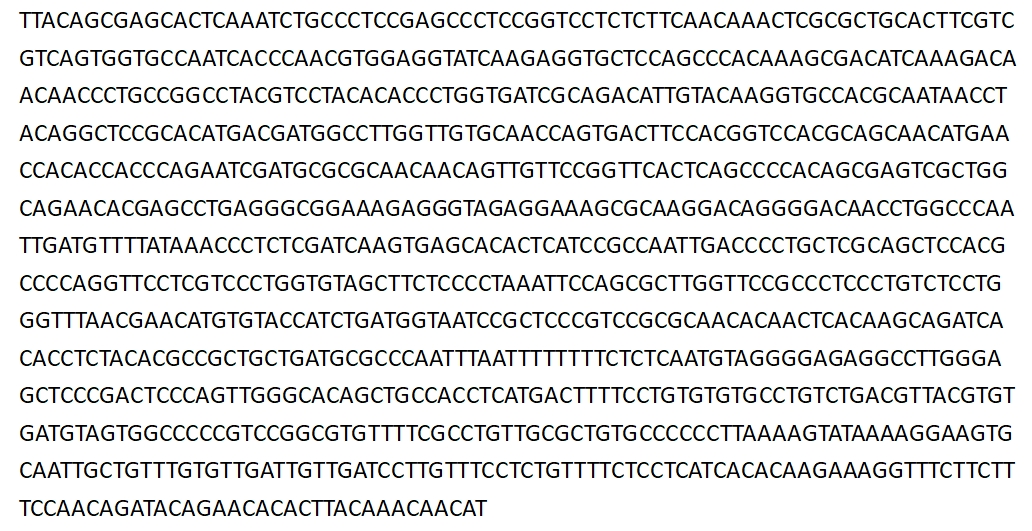

Supplement: Supplementary file 1 [file Supplementary_file_1.docx]
